# Editorial: Cancer Plasticity and the Microenvironment: Implications for Immunity and Therapy Response

**DOI:** 10.3389/fonc.2019.00276

**Published:** 2019-05-07

**Authors:** Andreas Behren, Erik W. Thompson, Robin L. Anderson, Petranel T. Ferrao

**Affiliations:** ^1^School of Cancer Medicine, La Trobe University, Heidelberg, VIC, Australia; ^2^Cancer Immunobiology Program, Olivia Newton-John Cancer Research Institute, Heidelberg, VIC, Australia; ^3^Institute of Health and Biomedical Innovation and School of Biomedical Sciences, Queensland University of Technology, and The Translational Research Institute, Brisbane, QLD, Australia; ^4^Translational Breast Cancer Program, Olivia Newton-John Cancer Research Institute, Heidelberg, VIC, Australia; ^5^Clinical Pathology and Surgery, The University of Melbourne, Melbourne, VIC, Australia

**Keywords:** immune evasion, therapy resistance, epithelial-to-mesenchymal transition (EMT), immunotherapy, tumor microenvironment (TME), heterogeneity, cancer plasticity, immune checkpoint inhibitors

Over the last decade, our understanding of how cancer cells interact with their microenvironment has grown exponentially. It has become evident that a complex interplay exists between malignant cells and benign host cells. The cancer cells and the “normal” cells, especially the immune compartment, undergo constant co-evolution to dynamically “shape” each other. Early attempts to utilize immune cells to favor anti-tumor responses date back more than a hundred years, when William Coley used bacteria to evoke immune responses in cancer patients, however, the results were controversial. Now, in the twenty-first century, “evading the immune system” has been recognized as a key hallmark of cancer (1).

The importance of the immune system in the recognition and clearance of cancer has been illustrated by the unprecedented success of immune checkpoint therapies in the treatment of multiple cancers and the durable responses observed in patients with late-stage disease (2, 3). Presently, clinical success has outpaced scientific understanding despite an ever increasing number of publications on a multitude of factors and pathways contributing to therapy response and resistance. What is clear, however, is that cancer cell plasticity—the ability of cancer cells to alter their phenotype or function—is linked to tumor cell dormancy, tumor progression, metastatic processes and treatment resistance (4). This can affect clonal selection and is implicated in immune evasion, thus influencing response to immunotherapy and patient outcome (5, 6). Cancer plasticity can be induced by a multitude of factors, most notably the tumor microenvironment (TME) (7). In this research topic, we have focused on plasticity within the tumor in the host TME, and various selective pressures that can dictate plasticity, or be influenced by it, particularly treatment responses.

Various features that are components of the TME have been recognized as potential prognostic and predictive biomarkers. The presence of tumor-infiltrating lymphocytes has been shown to correlate with improved survival (8), and this has led to the establishment of the biopsy-based immunoscore for colorectal cancer risk assessment (9). While the main focus of these studies was on the presence of T cells, the impact of several other immune cell types within the tumor microenvironment has also been reported (10). Using a retrospective correlative study of 269 triple-negative breast cancers (TNBCs), Yeong et al. reported that the density of plasma cells within TNBC tumors had a significant association with disease-free survival rates, and high expression of IgG genes was associated with improved survival outcomes. Through analysis of publicly available datasets for patients with hepatocellular carcinoma (HCC) and known outcomes, Shrestha et al. assessed immune modulators as potential biomarkers, highlighting that PD-L1 expression was closely associated with epithelial-to-mesenchymal transition (EMT) marker expression and acted as prognostic factor for poor survival in the high-risk patient group.

Tumor cells are known to exert an immune-suppressive or immune-evasive phenotype through various mechanisms, and the crosstalk between TME and tumor is now a major focus of research for understanding and exploiting the critical role of the immune system. Weidenfeld and Barkan highlight the role of EMT and its reverse process, mesenchymal-to-epithelial transition (MET) in tumor dissemination and dormancy. They discuss how EMT may lead to the acquisition of cancer stem cell-like traits in tumors, which can change the immune-regulating properties of these cancer cells, although the effect on immunotherapy outcomes is unclear. Poltavets et al. review how the extracellular matrix (ECM) can influence cancer cell plasticity, discussing the implications for immunotherapy and the potential to exploit targeting ECM regulators as novel therapeutic strategies. Ham et al. present data indicating that exosomes secreted by breast cancer cells can skew macrophage polarization toward a pro-tumoral M2 phenotype partially *via* gp130/STAT3 signaling, suggesting that the exosomes can enhance the immune-suppressive activity of macrophages. Hamilton et al. describe how down-regulation of cyclin-dependent kinase (CDK) inhibitor 1 (p21CIP1) in cancer cells by the transcription factor brachyury leads to less stable CDK1 and renders the tumor cells more resistant to chemotherapy and immune-mediated cytotoxicity.

The authors also speculate that the same mechanism may drive EMT, which in turn may influence tumor immunogenicity.

Heterogeneity and plasticity in the tumor, as well as within the TME, can affect treatment outcomes. This has been exemplified recently by tumors that are responsive/“hot” or resistant/“cold” to immunotherapies. A similar concept regarding therapeutic resistance in melanoma was reviewed by Ahmed and Haass. They highlight that specific gene expression patterns known to dictate cell phenotype and function could also be influenced by the microenvironmental conditions that are selective for subpopulations differing in proliferation rates, invasiveness and drug responsiveness. Tissue-specific TMEs at different anatomical locations can regulate tumor growth, determine metastatic progression and impact on the outcome of therapy responses as reviewed by Oliver et al. suggesting that the organ site of metastasis can also influence immunotherapy outcomes due to differences in the local TMEs.

One of the main mediators of immune stimulation following immunogenic cell death (ICD) is the type I interferons. Budhwani et al. review our current knowledge about how type 1 interferons assist in mounting an immune response, but also note that long term, chronic exposure to type I interferons may diminish the efficacy of radiotherapy and chemotherapy. Conversely, a loss of interferon signaling can lead to resistance to immunotherapy, similar to defects in the IFNγ signaling cascade, which can lead to resistance to anti-PD-1 therapies (11). Alavi et al. demonstrated that only 33% of a large panel of melanoma cell lines with diverse mutational drivers had a strong INFγ response, and displayed induction of all measured targets. The importance of these signaling pathways and the influence of the TME on signaling and gene expression patterns may, at least partially, explain why response to immunotherapy is restricted to subsets of patients.

More recently, additional tumor extrinsic features including the patient's innate immunity, extent and duration of inflammation, balance of the microbiome and even stress levels, are thought to influence patient outcomes. Andrews et al. review various factors that influence the “visibility” of the tumor to the immune system and how they together determine the susceptibility of the tumor to immune attack. They highlight the central role of the gut microbiome in influencing the overall immune set-point (12), through diverse effects on local and systemic inflammatory processes, to influence disease and treatment outcomes. As immune checkpoint blockade treatments are effective only in subsets of patients and can lead to severe immune-related adverse events, it is important to identify which patients are most likely to benefit from these treatments, and also those at risk of developing complications. In order to predict which patients will develop adverse effects to treatment before toxicity becomes clinically evident, Da Gama Duarte et al. reported the potential use of a protein array to capture auto-antibodies from a cohort of melanoma patients treated with immunotherapy.

As a high proportion of patients do not respond to current immunotherapies, novel targets that can enhance immunogenicity could be clinically beneficial. Fan et al. review the potential of retinoic acid receptor-related orphan receptors to directly affect tumor cell behavior, including their ability to directly influence immune cells, given their expression by regulatory T cells and other immune cell subsets. Effective immunotherapy requires cancer cells to be recognized as foreign by the immune system, triggering the initiation of a directed immune-response (13). Many current treatment approaches, either as mono-therapy or combination therapy, aim to initiate a strong and broad immune response that results in long-lasting anti-cancer immunity. Cruickshank et al. review the data showing that immunogenic cell death (ICD) leads to immune stimulation that is epigenetically regulated, and propose that epigenetic drugs like HDAC inhibitors could be used to modify ICD. Poh and Ernst review the role of macrophages, a key component of the TME that orchestrate various aspects of immunity to regulate tumor progression. They discuss the targeting of tumor-associated macrophages (TAMs) as anti-cancer treatment strategies, evaluating the contribution of macrophages in moderating the effectiveness of current therapies and the challenges for successfully incorporating these strategies in cancer treatment regimens.

We currently face several challenges for the clinical implementation of combination therapies as effective treatment strategies for cancer. This second edition of our Research Topic on Cancer Plasticity and the Microenvironment builds upon our first edition, Cellular and Phenotypic Plasticity in Cancer, by expanding the focus to aspects of immunity and therapy responses across multiple cancer types. Our topic emphasizes various aspects of cancer plasticity that are highly pertinent to the incorporation and effective use of immune-modulating drugs in conventional cancer treatment regimens. As we embark on an exciting and promising era of multi-modal precision therapy, we have highlighted here some relevant points for consideration in our efforts to improve cancer treatment strategies for better patient outcomes.

## Author Contributions

All authors listed have made a substantial, direct and intellectual contribution to the work, and approved it for publication.

### Conflict of Interest Statement

The authors declare that the research was conducted in the absence of any commercial or financial relationships that could be construed as a potential conflict of interest.
